# La tuberculose abdominale: une expérience de 13 ans

**DOI:** 10.11604/pamj.2025.51.75.47992

**Published:** 2025-07-14

**Authors:** Fatma Medhioub Kaaniche, Farah Zouari, Salma Jerbi, Yoser Ben Taher, Arij Abdellatif, Ines Dahech, Wiem Feki, Zina Hakim, Dorsaf Dlensi, Rania Allala

**Affiliations:** 1 Service Universitaire de Réanimation, Hôpital Régional Mahres, Faculté de Médecine de Sfax, Université de Sfax, Sfax, Tunisie,; 2 Service de Radiologie, Centre Hospitalier Universitaire Hédi Chaker, Faculté de Médecine de Sfax, Université de Sfax, Sfax, Tunisie,; 3 Unité de Médecine de Travail, Hôpital Régional Mahres, Faculté de Médecine de Sfax, Université de Sfax, Sfax, Tunisie

**Keywords:** Tuberculose abdominale, diagnostic, prise en charge, Abdominal tuberculosis, diagnosis, management

## Abstract

La tuberculose abdominale (TBA) est une forme rare mais grave de tuberculose extra-pulmonaire. Nous avons mené une étude prospective descriptive, incluant tous les patients hospitalisés à l´Hôpital régional de Mahres, pour une tuberculose abdominale entre janvier 2012 et décembre 2024. Nous avons inclus 14 patients. Les facteurs de risque comprenaient des antécédents personnels de tuberculose (28,5%), familiaux (21,4%), le diabète sucré (21,4%), la consommation de lait non pasteurisé (28,5%) et le contact avec des animaux (21,4%). Les principales localisations étaient péritonéales (42,8%) et intestinale (42,8%), avec atteinte ganglionnaire mésentérique dans 21,4% des cas. Une atteinte extra-abdominale était présente chez 35,7% des patients. Le diagnostic reposait sur des méthodes microbiologiques (42,8%), histologiques (35,7%) et /ou cliniques (21,4%). Tous les patients ont reçu une polychimiothérapie d´une durée médiane de 11 mois, bien tolérée sauf dans 14,2% des cas. La tuberculose abdominale reste une entité rare, aux formes cliniques variées et souvent trompeuses, soulignant l'importance d´une suspicion précoce et d´une prise en charge multidisciplinaire.

## Abstract (en)

Abdominal tuberculosis (ATB) is a rare but serious form of extrapulmonary tuberculosis. We conducted a prospective descriptive study including all patients hospitalized for abdominal tuberculosis at Mahres Regional Hospital between January 2012 and December 2024. Fourteen patients were included. Risk factors included a personal history of tuberculosis (28.5%), family history (21.4%), diabetes mellitus (21.4%), consumption of unpasteurized milk (28.5%), and contact with animals (21.4%). The main sites of involvement were peritoneal (42.8%) and intestinal (42.8%), with mesenteric lymph node involvement observed in 21.4% of cases. Extra-abdominal tuberculosis was present in 35.7% of patients. The diagnosis was based on microbiological methods (42.8%), histological findings (35.7%), and/or clinical features (21.4%). All patients received multi-drug anti-tuberculosis therapy for a median duration of 11 months, which was well tolerated except in 14.2% of cases. Abdominal tuberculosis remains a rare entity with diverse and often misleading clinical presentations, underscoring the importance of early clinical suspicion and multidisciplinary management.

## Introduction

La tuberculose est un problème majeur de santé publique dans le monde, avec une augmentation constante du nombre de cas ces dernières années. Son impact varie selon les pays, avec une estimation de 10,6 millions de nouveaux cas en 2021, marquant une augmentation de 4,5% par rapport à 2020 [1]. Cette maladie infectieuse peut toucher divers organes, bien que l'atteinte pulmonaire reste la plus fréquente [2]. Cependant, les formes extra-pulmonaires de la tuberculose sont de plus en plus fréquentes, posant ainsi un défi diagnostique et thérapeutique [3].

Cette étude vise à analyser les caractéristiques cliniques, biologiques, bactériologiques, thérapeutiques et évolutives des patients atteints de tuberculose abdominale dans la région de Mahres et ses provinces. Une meilleure compréhension de ces éléments contribuerait à un diagnostic précoce et à une prise en charge optimale des patients, réduisant ainsi les risques de complications et garantissant une meilleure issue thérapeutique.

## Méthodes

**Type d'étude:** nous avons mené une étude prospective descriptive incluant tous les patients hospitalisés à l'hôpital régional de Mahres pour une tuberculose extra-pulmonaire (TEP) entre janvier 2012 et décembre 2024.

**Critères d'inclusion:** nous avons analysé les caractéristiques cliniques, biologiques et évolutives des patients hospitalisés à l'hôpital régional de Mahres pour tuberculose abdominale (TAB). Les patients dont le diagnostic de TAB a été douteux n'ont pas été inclus.

**Critères d'exclusion:** nous avons exclu de notre étude les patients chez qui la collecte des données a été incomplète.

**Collecte des données:** le diagnostic de la TBA reposait sur des preuves microbiologiques, représentées par un frottis ou une culture positive de bacilles acido-alcoolo-résistants à partir d'un échantillon d'ascite ou de biopsie, et/ou sur des preuves histologiques, caractérisées par la présence de granulomes caséeux sur un prélèvement biopsique abdominal. Une confirmation microbiologique ou histologique d'une tuberculose, associée à des signes cliniques et radiologiques évocateurs de TBA, permettait d'établir le diagnostic. En l'absence de ces éléments, la tuberculose abdominale était cliniquement confirmée lorsqu'une présentation typique était associée à un test tuberculinique (TST) positif et à des examens diagnostiques négatifs. Une réponse favorable au traitement antituberculeux permettait également de confirmer le diagnostic, défini par la disparition des symptômes abdominaux et systémiques ainsi que par la normalisation des analyses biologiques. L'imagerie abdominale de contrôle ou la coloscopie de surveillance n'était pas systématiquement indiquée, sauf en cas d'absence d'amélioration sous traitement antituberculeux.

**Analyse statistique:** elle a été réalisée à l'aide du logiciel SPSS 20. Les variables catégorielles ont été exprimées en effectifs et en pourcentages. Lorsque la distribution était normale, les variables continues ont été exprimées sous forme de moyennes et d'écarts-types. En cas de distribution non normale, la médiane et l'intervalle interquartile ont été utilisés.

## Résultats

**Caractéristiques épidémiologiques des cas de tuberculose abdominale:** au cours de la période d'étude, nous avons recensé 14 patients atteints de tuberculose abdominale. L'âge moyen des patients était de 42 ± 13 ans. Parmi les 14 patients, 8 sont des hommes (57%) et 6 des femmes (43%) avec un sex-ratio de 1,33. Les antécédents les plus fréquents étaient la tuberculose personnelle (28,5%) (ganglionnaire chez 2 patients et pulmonaire chez 2 autres), suivie de la tuberculose familiale et du diabète sucré (21,4% chacun). Parmi les facteurs d'exposition identifiés, la consommation de lait non pasteurisé a été rapportée par 4 patients, représentant 28,5% des cas. Par ailleurs, 3 patients (21,4%) ont mentionné un contact étroit avec des animaux domestiques ou d'élevage.

**Circonstances de découverte:** parmi les 14 patients étudiés, plusieurs symptômes étaient fréquemment rapportés. La fièvre était le symptôme le plus courant, touchant 71,4% des patients (10 personnes). Elle était suivie de l´asthénie, signalée par 64,2% des patients (9 personnes). De plus, 57,1% des patients (8 personnes) souffraient d´une perte de l´appétit, tandis que 50% (7 personnes) présentaient une perte de poids significative. Enfin, les sueurs nocturnes étaient rapportées par 42,8% des patients (6 personnes) et une douleur abdominale par 28,5% des patients (4 personnes) sans autre manifestation digestive.

**Localisations de la tuberculose abdominale:** les localisations les plus fréquentes chez les patients étudiés sont principalement l'atteinte péritonéale dans 42,8% des cas (6 patients), l'ascite sèche chez 35,7% des patients (5 cas) et l'ascite humide chez 7,1% des patients (1 cas). L'atteinte intestinale a touché 42,8% des patients (6 cas), répartis entre une atteinte iléo-cæcale prédominante à 28,6% (4 cas) et une atteinte appendiculaire plus rare à 7,1% (1 cas).

L'atteinte ganglionnaire mésentérique était également présente chez 21,4% des patients (3 cas). Enfin, des atteintes hépatiques sont relevées chez 14,3% des patients (2 cas).

Les formes mixtes étaient observées chez 21,4% des patients (3 cas): atteinte péritonéale sèche et ganglionnaire (7,1%, 1 cas), atteinte iléo-cæcale et ganglionnaire (7,1%, 1 cas), ainsi qu´atteinte péritonéale humide et iléo-cæcale (7,1%, 1 cas) (Tableau 1).

**Tableau 1:** sites atteints des cas de tuberculose abdominale

Localisation	Type d'atteinte	Nombre de patients	Fréquence (%)
**Péritonéale**	Ascite sèche	5	35.7
	Ascite humide	1	7.1
**Intestinale**	Atteinte iléo-cæcale	4	28.6
	Atteinte appendiculaire	1	7.1
**Ganglionnaire**	Atteinte mésentérique	3	21.4
**Hépatique**	Atteinte du foie	2	14.2
**Formes mixtes**	Péritonéale sèche +ganglionnaire	1	7.1
	Iléo-cæcale+ganglionnaire	1	7.1
	Péritonéale humide+iléo-cæcale	1	7.1

**Autres localisations de la tuberculose:** dans cette étude, la tuberculose abdominale a été fréquemment associée à d'autres formes de tuberculose, illustrant ainsi des cas de tuberculose multifocale, où plusieurs organes sont simultanément atteints. Deux patients (14,2%) ont présenté une atteinte pulmonaire associée. Un patient (7,1%) a montré une atteinte ostéoarticulaire. De plus, deux patients (14,2%) ont eu une tuberculose urogénitale et deux autres (14,2%) ont présenté une tuberculose des ganglions lymphatiques extra-abdominaux.

**Données paracliniques:** les analyses biologiques ont révélé des niveaux élevés de protéine C-réactive (CRP) dans 8 cas (57,1%), ainsi qu'une vitesse de sédimentation des érythrocytes (VSE) accélérée dans 7 cas (50%). Une anémie a été observée dans 9 cas (64,2%). L'intradermoréaction à la tuberculine (IDR) a révélé une réponse positive chez 10 patients (71,4%), avec une induration supérieure à 10 mm. En revanche, 4 patients (28,5%) ont présenté un résultat négatif.

**Confirmation diagnostique:** le diagnostic a été confirmé principalement par des méthodes microbiologiques, chez 6 patients (42,8%) ayant obtenu une confirmation par *polymerase chain reaction* (PCR) ou culture du liquide d´ascite. L´examen histologique, basé sur l'analyse de biopsies ganglionnaires ou hépatiques, a permis de confirmer le diagnostic chez 5 patients (35,7%). Une preuve à la fois microbiologique et histologique a été obtenue dans 2 cas (14,2%). Enfin, 3 patients (21,4%) ont été diagnostiqués sur la base de critères cliniques, en l'absence de confirmation microbiologique ou histologique (Tableau 2).

**Tableau 2:** méthodes de confirmation des cas de tuberculose abdominale

Méthode de diagnostic	Nombre de patients	Fréquence (%)
Confirmation microbiologique	6	42.8
Confirmation histologique	5	35.7
Confirmation microbiologique et histologique	2	14.2
Diagnostic basé sur des critères cliniques	3	21.4

### Prise en charge thérapeutique

*Traitement antituberculeux:* la majorité des patients, soit 12 patients (85,7%), ont reçu une polychimiothérapie standard, combinant plusieurs antituberculeux conformément aux recommandations en vigueur. Parmi eux, 8 patients (57,1%) ont reçu la forme dissociée (médicaments administrés séparément), tandis que 4 patients (28,6%) ont bénéficié de la forme combinée (comprimés fixes combinés). Le protocole standard comprenait une phase initiale intensive de 2 mois, associant isoniazide (5 mg/kg/j), rifampicine (10 mg/kg/j), pyrazinamide (25 mg/kg/j) et éthambutol (15 mg/kg/j), suivie d'une phase de continuation de 4 à 10 mois avec isoniazide et rifampicine aux mêmes doses. En revanche, 2 patients (14,2%) ont bénéficié d'un traitement adapté en raison d'effets secondaires nécessitant une modification du protocole initial. La durée médiane du traitement antituberculeux était de 11 mois [10 à 15 mois].

*Corticothérapie:* trois patients (21,4%) ont reçu une corticothérapie adjuvante. Les principales indications étaient une compression digestive causée par des masses ganglionnaires chez un patient et un épanchement péritonéal abondant avec réaction inflammatoire importante chez deux autres. Le protocole administré consistait en Prednisone à une dose initiale de 0,5 à 1 mg/kg/j, avec une réduction progressive sur une période de 4 à 8 semaines, selon la réponse clinique et l´évolution des symptômes.

**Evolution:** parmi les patients sous traitement antituberculeux, 2 patients (14,2%) ont signalé des effets indésirables cliniques, type des nausées et des vomissements. Un patient (7,1%) a présenté une cytolyse hépatique. Une leucopénie a été observée chez un autre (7,1%). De plus, un patient (7%) a développé une neuropathie optique. Au cours du suivi, 2 patients (14,2%) ont présenté une occlusion intestinale, complication majeure nécessitant une prise en charge spécifique dont un patient (7,1%) a conservé des adhérences péritonéales. Aucune rechute n'ayant été observée et aucun décès n'ayant été rapporté.

## Discussion

**Sites affectés:** la tuberculose abdominale est une forme complexe et polymorphe de tuberculose extra-pulmonaire, pouvant toucher diverses structures du système digestif, le péritoine, les ganglions lymphatiques ainsi que plusieurs organes viscéraux. L'analyse des études comparatives permet de mettre en lumière la variabilité des localisations et des formes cliniques de cette pathologie. Parmi les quatre formes principales: péritonéale, intestinale, viscérale et ganglionnaire, la tuberculose péritonéale semble être la plus fréquemment rapportée (Tableau 3). Dans notre étude, elle représente 42,8% des cas, avec une prédominance du type ascitique sec (35,7%) par rapport au type humide (7,1%). Cette prédominance est nettement supérieure à celle observée chez Cho *et al*. (20,1%), Tan *et al*. (22,8%) et inférieure à celle rapportée par Fatma *et al*. (68,6%) [4-6].

**Tableau 3:** sites affectés des cas de tuberculose abdominale dans la littérature

Localisation	Type d'atteinte	Cho *et al*. (%) (4)	Tan *et al*. (%) (5)	Fatma *et al*. (%) (6)	Notre étude (%)
Péritonéale		20.1	22.8	68.6	42.8
	Ascite humide	75.7	-	60.5	7.1
	Ascite sèche	24.3	-	8.1	35.7
Intestinale		49.6	57.9	18.6	28.6
-	Atteinte iléo-cæcale	67.1	28.1	10.5	28.6
-	Atteinte jéjunale	9.2	8.8	4.7	-
-	Atteinte appendiculaire	-	-	3.5	7.1
Viscérale		16.5	19.3	14	14.2
-	Atteinte hépatique	10.3	3.5	7	14.2
-	Atteinte splénique	13.8	12.3	5.8	-
-	Atteinte surrénalienne	3.4	-	1.1	-
Ganglionnaire		5	42.1	14	21.4
Formes mixtes	Péritonéale+gastro-intestinal	33.3	-	3.5	7.1
-	Péritonéale+viscérale	16.7	-	3.5	-
-	Péritonéale + ganglionnaire	-	-	2.3	7.1
-	Gastro-intestinal+ ganglionnaire	3.8	-	3.5	-
-	Gastro-intestinal+ viscérale	-	-	1.1	-
-	Viscérale+ ganglionnaire	16.7	-	1.1	7.1

La tuberculose intestinale, bien que moins fréquente dans notre étude (35,7%) et celle de Fatma *et al*. (18,6%) [6], est une forme majeure dans les études de Cho *et al*. (49,6%), Tan *et al*. (57,9%) [4,5]. Les localisations iléo-caecales prédominent (28,6%) dans notre étude, sont suivies des atteintes appendiculaires (7,1%). L'atteinte intestinale est souvent difficile à diagnostiquer en raison de la présentation clinique peu spécifique et de l'absence de signes radiologiques caractéristiques, ce qui peut expliquer son identification plus fréquente dans des centres tertiaires spécialisés.

Les formes viscérales de la tuberculose abdominale, bien qu'assez rares, restent significatives. Elles incluent des localisations hépatiques (7%), spléniques (5,8%) et surrénaliennes (1,1%) dans l'étude de Fatma *et al*. [6]. La tuberculose hépatique est particulièrement difficile à diagnostiquer, nécessitant souvent des biopsies hépatiques pour confirmation. Les atteintes ganglionnaires, quant à elles, montrent une prévalence plus élevée chez Tan *et al*. (42,1%) par rapport à notre étude (14%) et à celle de Cho *et al*. (5%) [4].

Enfin, des formes mixtes associant plusieurs localisations sont décrites. Les combinaisons les plus fréquentes incluent les associations péritonéales et intestinales (3,5%), péritonéales et viscérales (3,5%), ainsi que des formes triples rares comme la coexistence de localisations viscérales, ganglionnaires et intestinales (1,1%) [6]. Ces associations complexes témoignent de la nature souvent disséminée de la maladie.

**Confirmation du diagnostic:** la tuberculose abdominale repose sur une approche multidisciplinaire associant méthodes microbiologiques, analyses histopathologiques et critères cliniques. Dans notre étude, la confirmation microbiologique a été obtenue chez 42,8% des patients (6 cas), grâce à des techniques modernes comme la culture des prélèvements et la PCR. Cette approche a permis une détection précise du complexe *Mycobacterium tuberculosis*. L'examen histopathologique, quant à lui, a permis de poser le diagnostic chez 35,7% des patients (5 cas), en identifiant des granulomes épithélioïdes caséeux caractéristiques de l'infection tuberculeuse. Une double confirmation microbiologique et histologique a été observée dans 14,2% des cas (2 patients), renforçant la certitude diagnostique. Enfin, 21,4% des patients (3 cas) ont été diagnostiqués sur la base de critères cliniques seuls, faute de preuves microbiologiques ou histologiques.

Ces résultats présentent certaines similitudes, mais aussi des divergences par rapport aux données de la littérature. Sanai et Bzeizi [7], rapportent une sensibilité limitée de la culture, comprise entre 20 et 40%, en raison de la lente croissance du bacille tuberculeux. Les prélèvements d'ascite restent les plus fréquents, bien que souvent pauci-bacillaires, limitant la sensibilité des cultures. Roberts *et al*. [8] soulignent l'importance de l'histopathologie à partir de biopsies de sites péritonéaux ou intestinaux, avec une sensibilité proche de 80% lorsqu'un granulome caséeux est identifié.

Wu *et al*. [9] mettent en avant la nécessité des prélèvements guidés par imagerie pour optimiser les chances diagnostiques, en particulier pour les biopsies des ganglions mésentériques et des épaississements pariétaux intestinaux. Cependant, malgré ces avancées, les diagnostics basés uniquement sur des données cliniques restent fréquents, notamment dans les contextes de ressources limitées, comme en témoigne notre taux de 21,4%. Cette observation reflète les défis persistants pour obtenir une preuve définitive de la tuberculose abdominale, soulignant l'importance d'une stratégie diagnostique intégrée et adaptée à chaque contexte clinique.

### Prise en charge thérapeutique

*Traitement antituberculeux:* le traitement de la tuberculose abdominale repose essentiellement sur une polychimiothérapie antituberculeuse associant plusieurs médicaments. Dans notre étude, 85,7% des patients (12 cas) ont reçu un protocole standard comprenant une phase initiale intensive de deux mois à base d'isoniazide, rifampicine, pyrazinamide et éthambutol, suivie d'une phase de consolidation de sept à dix mois avec isoniazide et rifampicine. La durée médiane du traitement était de 11 mois [10 à 15 mois]. Deux patients (14,2%) ont nécessité une adaptation thérapeutique en raison d'effets indésirables, soulignant l'importance d'un suivi clinique et biologique régulier pour surveiller les effets secondaires liés aux antituberculeux.

Les données de la littérature corroborent nos résultats. Sanai et Bzeizi [7], recommandent une durée de traitement de 9 à 12 mois pour les formes complexes de tuberculose abdominale. Une revue récente d'Evans *et al*. [10] souligne l'importance d'une approche personnalisée, notamment chez les patients présentant des localisations multiples ou une atteinte viscérale sévère. De leur côté, Pusch *et al*. [11] recommandent une thérapie longue pour prévenir les récidives, tout en ajustant la durée en fonction de la réponse clinique et biologique du patient.

Toutefois, certains travaux plus récents, comme ceux de Jullien *et al*. [12] plaident pour une réduction de la durée du traitement à six mois dans les formes simples de tuberculose abdominale. Cette approche, bien qu'efficace dans certaines conditions, reste controversée en raison du risque potentiel de rechutes. Sharma *et al*. [13] recommandent un suivi régulier des niveaux de CRP pour évaluer la réponse au traitement et ajuster la durée de la thérapie.

Dans notre série, l'adhésion majoritaire au schéma thérapeutique classique a permis d'obtenir une évolution favorable sans rechute. L'adaptation du traitement chez deux patients démontre cependant la nécessité d'une approche flexible et individualisée, notamment pour gérer les toxicités médicamenteuses. Ces résultats s'alignent sur les recommandations internationales tout en soulignant l'importance d'un suivi rigoureux et de critères biologiques, pour optimiser la prise en charge thérapeutique [14].

*Traitement par corticostéroïdes:* l'utilisation des corticostéroïdes comme traitement adjuvant dans la tuberculose abdominale soulève des interrogations cliniques quant à leurs bénéfices et leurs risques. Dans notre étude, trois patients (21,4%) ont bénéficié d'une corticothérapie adjuvante. Les objectifs thérapeutiques étaient de réduire l´intensité de la réponse inflammatoire, de limiter la formation d'adhérences intra-abdominales et d'accélérer la résolution des symptômes.

La littérature appuie ces indications sous certaines conditions. Soni *et al*. [15], dans une méta-analyse regroupant plusieurs études internationales, ont mis en évidence l'efficacité des corticostéroïdes pour diminuer les complications péritonéales graves et améliorer les taux de rémission clinique. Leur analyse conclut que l'adjonction de corticoïdes peut favoriser une résolution plus rapide des masses inflammatoires abdominales. Cependant, ils mettent en garde contre le risque potentiel d'immunosuppression qui pourrait favoriser une dissémination tuberculeuse ou retarder la clairance bactérienne.

Calin *et al*. [16], dans une étude menée dans deux hôpitaux parisiens, rapportent une prescription plus fréquente de corticostéroïdes (30% des cas) pour les formes compliquées de la tuberculose abdominale, notamment chez les patients avec une ascite massive ou des atteintes péritonéales étendues. Les résultats suggèrent une nette amélioration symptomatique chez les patients sous traitement adjuvant, bien que certains aient développé des infections secondaires.

Cherian *et al*. [17] insistent quant à eux sur la nécessité de bien sélectionner les patients, en réservant cette approche aux cas sévères, notamment ceux présentant une réponse inflammatoire exacerbée. Leur étude montre une meilleure tolérance au traitement global chez les patients ayant reçu une corticothérapie contrôlée.

## Conclusion

Cette étude met en évidence les défis diagnostiques et thérapeutiques posés par la tuberculose abdominale. L'association fréquente avec d'autres formes de tuberculose, la diversité des manifestations cliniques et la nécessité d'un diagnostic de certitude par des examens microbiologiques ou histologiques justifient une prise en charge multidisciplinaire. Une meilleure sensibilisation des cliniciens aux facteurs de risque et aux manifestations atypiques de la TBA est essentielle pour limiter les retards diagnostiques et optimiser le traitement. Enfin, la prévention repose sur le contrôle des facteurs de transmission, notamment l'amélioration des conditions sanitaires et l'éducation sur les risques liés à la consommation de produits non pasteurisés et au contact avec des animaux.

### Etat des connaissances sur le sujet

- La tuberculose abdominale est une forme peu fréquente mais potentiellement grave de tuberculose extra-pulmonaire;
- Son diagnostic est souvent retardé en raison de signes cliniques polymorphes et non spécifiques;
- Les méthodes de confirmation diagnostique incluent les examens microbiologiques, histologiques et l'évaluation clinique.

### Contribution de notre étude à la connaissance

- Une analyse prospective de 14 cas de tuberculose abdominale hospitalisés sur 13 ans dans un hôpital régional tunisien;
- L'identification des facteurs de risque spécifiques dans ce contexte: antécédents tuberculeux, diabète, consommation de lait non pasteurisé;
- Une description des localisations cliniques, des approches diagnostiques utilisées et de la tolérance au traitement antituberculeux.
